# Long-term cardiovascular mortality risk in patients with bladder cancer: a real-world retrospective study of 129,765 cases based on the SEER database

**DOI:** 10.3389/fcvm.2023.1142417

**Published:** 2023-11-09

**Authors:** Jia Liao, Zihua Zhou

**Affiliations:** Department of Cardiology, Union Hospital, Tongji Medical College, Huazhong University of Science and Technology, Wuhan, China

**Keywords:** bladder cancer, cardiovascular mortality, cardio-oncology, SEER database, competing risk model, nomogram

## Abstract

**Introduction:**

Among 28 cancer types, bladder cancer (BC) patients have the highest risk of dying from cardiovascular disease (CVD). We aimed to identify the independent risk factors and develop a novel nomogram for predicting long-term cardiovascular mortality in patients with BC.

**Methods:**

We extracted data from the Surveillance, Epidemiology, and End Results (SEER) database for patients diagnosed with bladder cancer (BC) between 2000 and 2017. The cumulative incidence function (CIF) was computed for both CVD-related death and other causes of death. Then we performed univariate and multivariate analyses to explore the independent risk factors and further develop a novel nomogram to predict cardiovascular mortality at 5- and 10-year for patients with BC by using the Fine-Gray competing risk model. The efficacy of the developed nomogram was assessed by the concordance index (C-index), receiver operating characteristic (ROC) curve, calibration curve, and decision curve analysis (DCA).

**Results:**

A total of 12,9765 patients were randomly divided into training (*n* = 90,835, 70%), and validation (*n* = 38,930, 30%) cohorts. During the follow-up period, 31,862 (46.4%) patients died from BC, and 36793 (53.6%) patients died from non-BC, of which CVD-related death accounted for 17,165 (46.7%), being the major cause of non-cancer deaths. The multivariate analysis showed that age, sex, race, marital status, histologic type, tumor grade, summary stage, and chemotherapy were independent risk factors of CVD-related death in BC patients. The nomogram based on the above eight factors showed good discrimination power, excellent consistency, and clinical practicability: (1) the areas under the curve of the ROC for 5- and 10-year CVD-related death of 0.725 and 0.732 in the training cohort and 0.726 and 0.734 in the validation cohort; (2) the calibration curves showed that the prediction probabilities were basically consistent with the observed probabilities; (3) the DCA curves revealed that the nomogram had high positive net benefits.

**Discussion:**

To our knowledge, this was the first study to identify the independent risk factors and develop a novel nomogram for predicting long-term cardiovascular mortality in patients with BC based on the competing risk model. Our results could help clinicians comprehensively and effectively manage the co-patient of BC and CVD, thereby reducing the risk of cardiovascular mortality in BC survivors.

## 1. Introduction

Bladder cancer (BC) is the most frequently diagnosed malignant tumor in the urinary system and ranks among the top ten most prevalent malignancies worldwide. As estimated by GLOBOCAN in 2020, this disease is projected to account for 573,278 new cases and 212,536 BC-related deaths, resulting in a significant disease burden, increased healthcare costs, and diminished health-related quality of life (1, 2). With the improvement of modern medical care, the life expectancy of cancer patients has increased. The current study reports a higher proportion of non-cancer deaths among all BC patients, with cardiovascular death being one of the primary causes of non-cancer deaths (3–8).

Cardiovascular disease (CVD) represents the leading cause of mortality worldwide (9, 10). More and more evidences indicate that tumors and CVD share several high-risk factors, such as obesity, hyperglycemia, inflammation, hormone replacement therapy, smoking, and lower socioeconomic status, requiring common target interventions to prevent the onset and progression of tumors and CVD (11–14). Many cancer therapies, including chest irradiation, chemotherapy, and immunotherapy, exhibit varying degrees of cardiovascular toxicity (15, 16). Therefore, the incidence of concomitant or secondary CVD in cancer survivors is increasing every year, leading to the formation of a new discipline: cardio-oncology.

A previous observational study of Surveillance, Epidemiology, and End Results (SEER) data reported that BC patients have the highest risk of dying from CVD among 28 cancer types, with 19.4% of BC patients had died from CVD (4). In addition, elderly patients with BC demonstrated an elevated risk of CVD-related death as compared to the general population (17). Approximately 5–10 years following diagnosis, CVD-related death superseded BC as the primary cause of death among older BC patients, particularly among subgroups of patients with localized and low-grade tumors (8).

In our study, we aimed to conduct a population-based analysis of a cohort of BC patients in the SEER database to identify the independent risk factors associated with CVD-related death, and subsequently, to build and validate a scoring system to predict the long-term cardiovascular mortality risk of BC patients. Cardio-oncology research studies often necessitate consideration of potential competing risks, wherein the incidence of other events (e.g., cancer-related death) may preclude the primary event of interest (e.g., cardiovascular outcome) (18). As such, our work is all based on the method of the competing risk model. Our results could help clinicians comprehensively and effectively manage the co-patient of BC and CVD and reduce the risk of cardiovascular mortality in BC survivors.

## 2. Materials and methods

### 2.1. Data source and patient selection

Data from the Surveillance, Epidemiology, and End Results (SEER) database (http://seer.cancer.gov/) were used in our study.The SEER program of the National Cancer Institute is a network of population-based incident tumour registries, covering 28% of the US population, including incidence, survival, and treatment (19). Given that our study solely utilized identified data from the SEER database and did not involve the direct participation of patients, institutional review board approval and informed consent were deemed unnecessary for this investigation. The Strengthening the Reporting of Observational Studies in Epidemiology (STROBE) guidelines were adhered to whilst conducting this research (20).

Patients diagnosed with BC from 2000 to 2017 from 17 population-based cancer registries were included in our study. The inclusion criteria for our study were as follows: (1) case selection based on site and morphology, with primary site labeled as “C67.0–9”; (2) availability of active follow-up information and defined causes of mortalities; (3) BC was the only one and primary cancer. Next, we collected the following informations of included patients: age, sex, race, marital status, primary site, histologic type, tumor grade, summary stage, surgery, radiotherapy, chemotherapy, survival months, and causes of death. Patients with incomplete data about any of the aforementioned variables were excluded.

### 2.2. Study outcomes

The primary outcome was death from CVD. As recorded in the SEER database, CVD-related death was defined by the following six causes: (1) disease of heart, (2) hypertension without heart disease, (3) cerebrovascular disease, (4) atherosclerosis, (5) aortic aneurysm and dissection, (6) other diseases of arteries, arterioles, and capillaries. Competing events against cardiovascular death in our study consisted of deaths from BC and other non-CVD related causes. Follow-up time was calculated from the initial diagnosis of BC until the patient was lost to follow-up, date of death, or final follow-up date.

### 2.3. Statistical analysis

The patients included in this study were randomly assigned to either training or validation cohort in a 7:3 ratio. Differences in baseline characteristics between the two groups were assessed using the chi-squared test. To assess the interaction between BC and CVD-related deaths, we calculated the cumulative incidence function (CIF) to determine the probabilities of each event at 5- and 10-year. Fine and Gray's subdistribution proportional hazards model was conducted on the training cohort to identify the independent risk factors of death from CVD and then construct a novel competing risk model to predict the probabilities of CVD-related death at 5- and 10-year for patients with BC. Then to measure the discriminative performance of the model, we calculated the bootstrap-corrected concordance index (C-index) and constructed the receiver operating characteristic (ROC) curves based on the area under the curve (AUC) values of the corresponding variables. The calibration curves and decision curve analysis (DCA) were performed to verify the consistency and investigate the clinical utilities and net benefits of this model.

All analyses were performed using SEER*Stat software (version 8.4.0.1, National Cancer Institute, Bethesda, MD, USA), Stata/MP software (version 16.0, Stata Corp, College Station, TX, USA), and R software (version 4.2.1; The R Foundation for Statistical Computing, Vienna, Austria). A two-sided *p*-value < 0.05 was considered statistically significant.

## 3. Results

### 3.1. Patients characteristics

In this study, a total of 129,765 eligible patients diagnosed with BC from the SEER database were randomly assigned to either training (*n* = 90,835, 70%) or validation (*n* = 38,930, 30%) cohort. Most of the patients were over 65 years old (67.5%), male (73.8%), white (89.7%), and married (62.6%). A larger proportion of patients were diagnosed with transitional cell carcinoma (95.8%), grade Ⅳ (32.7%), and *in situ* (47.3%). With regard to treatment, the majority of patients had undergone surgery (96.2%) and only a small number of patients had received radiotherapy (5.9%) or chemotherapy (21.1%). As for the causes of death, 31862 (46.4%) patients died due to BC, and 36793 (53.6%) patients died due to non-BC, of which CVD-related death accounted for 17165 (46.7%), being the major cause of non-cancer deaths.

No statistically significant differences in any of the analyzed variables, including age, sex, race, marital status, primary site, histologic type, tumor grade, summary stage, surgery, radiotherapy, chemotherapy, and causes of death, were observed between the training and validation cohorts (Table 1).

**Table 1:** The Baseline demographic and clinicopathological characteristics of the included BC patients**.**

Variables	Training cohort		Validation cohort		Total		*P* value
	90,835	70%	38930	30%	1,29,765	100%	
Age (years)							0.924
<64	29499	32.5%	12686	32.6%	42185	32.5%	
65–74	25605	28.2%	10919	28.0%	36524	28.1%	
75–84	24676	27.2%	10555	27.1%	35231	27.1%	
>85	11055	12.2%	4770	12.3%	15825	12.2%	
Sex							0.377
Male	67,130	73.9%	28,679	73.7%	95,809	73.8%	
Female	23,705	26.1%	10,251	26.3%	33,956	26.2%	
Race							0.323
White	81,494	89.7%	34,926	89.7%	1,16,420	89.7%	
Black	4,818	5.3%	2,122	5.5%	6,940	5.3%	
Others	4,523	5.0%	1,882	4.8%	6,405	4.9%	
Marital status							0.676
Married	56,786	62.5%	24,385	62.6%	81,171	62.6%	
Single/other	34,049	37.5%	14,545	37.4%	48,594	37.4%	
Primary site							0.868
C67.0-Trigone of bladder	6,048	6.7%	2,522	6.5%	8,570	6.6%	
C67.1-Dome of bladder	3,264	3.6%	1,416	3.6%	4,680	3.6%	
C67.2-Lateral wall of bladder	20,004	22.0%	8,698	22.3%	28,702	22.1%	
C67.3-Anterior wall of bladder	2,033	2.2%	883	2.3%	2,916	2.2%	
C67.4-Posterior wall of bladder	8,599	9.5%	3,677	9.4%	12,276	9.5%	
C67.5-Bladder neck	2,543	2.8%	1,122	2.9%	3,665	2.8%	
C67.6-Ureteric orifice	3,660	4.0%	1,560	4.0%	5,210	4.0%	
C67.7-Urachus	153	0.2%	72	0.2%	225	0.2%	
C67.8-Overlapping lesion of bladder	10,905	12.0%	4,678	12.0%	15,583	12.0%	
C67.9-Bladder, NOS	33,636	37.0%	14,302	36.7%	47,938	36.9%	
Histologic type							0.729
Transitional cell carcinoma	87,039	95.8%	37,308	95.8%	1,24,347	95.8%	
Squamous cell carcinoma	1,686	1.9%	705	1.8%	2,391	1.8%	
Adenocarcinoma	695	0.8%	318	0.8%	1,013	0.8%	
Other and unclassified carcinoma	1,415	1.6%	599	1.5%	2,014	1.6%	
Tumor grade							0.935
I	12,587	13.9%	5,443	14.0%	18,030	13.9%	
II	28,506	31.4%	12,183	31.1%	40,699	31.4%	
III	20,014	22.0%	8,553	22.0%	28,567	22.0%	
IV	29,728	32.7%	12,751	32.8%	42,479	32.7%	
Summary stage							0.278
In situ	42,890	47.2%	18,424	47.3%	61,314	47.3%	
Localized	35,837	39.5%	15,191	39.0%	51,028	39.3%	
Regional	7,679	8.5%	3,345	8.6%	11,024	8.5%	
Distant	4,429	4.9%	1,970	5.1%	6,399	4.9%	
Surgery							0.257
No	3,395	3.7%	1,506	3.9%	4,901	3.8%	
Yes	87,440	96.3%	37,424	96.1%	1,24,864	96.2%	
Radiotherapy							0.068
No	85,526	94.2%	36,553	93.9%	1,22,079	94.1%	
Yes	5,309	5.8%	2,377	6.1%	7,686	5.9%	
Chemotherapy							0.638
No	71,657	78.9%	30,756	79.0%	1,02,413	78.9%	
Yes	19,178	21.1%	8,174	21.0%	27,352	21.1%	
Causes of death							0.948
Death from BC	22,298	24.5%	9,564	24.6%	31,862	24.6%	
Death from CVD	11,997	13.2%	5,168	13.3%	17,165	13.2%	
Death from other causes	13,772	15.2%	5,856	15.0%	19,628	15.1%	
Total death	48,067	52.9%	20,588	52.9%	68,655	52.9%	

### 3.2. Cumulative incidence function survival analysis

**Figure 1:**
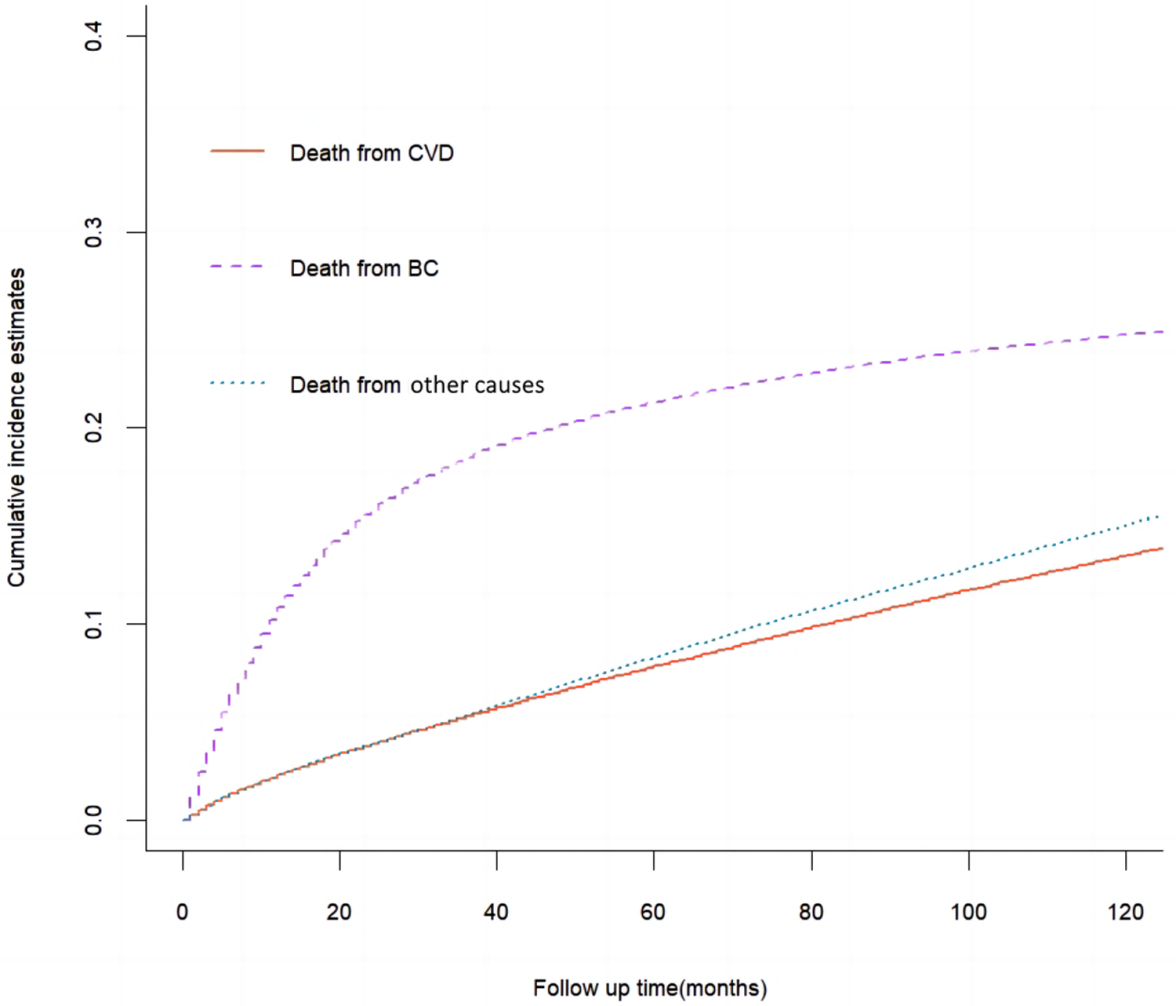
Cumulative incidence estimates of death causes among patients with bladder cancer (BC) in the whole cohort.

**Figure 2:**
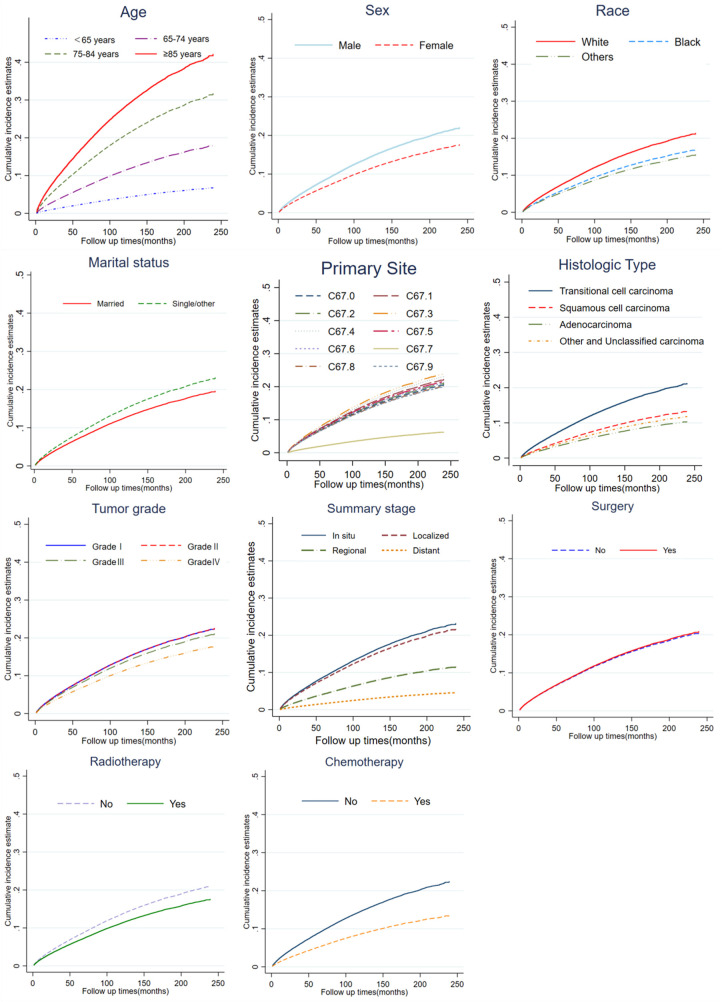
Cumulative incidence estimates of CVD-related death among patients with BC according to age, sex, race, marital status, primary site, histologic type, tumor grade, summary stage, surgery, radiotherapy and chemotherapy.

The results of cumulative incidence analysis showed that the 5-, and 10-year CIF of death due to BC were 21.3 and 24.8%, the 5-, and 10-year CIF of death due to CVD were 7.8 and 13.5%, and the 5-, and 10-year CIF of death due to non-CVD were 8.3 and 15.1%, respectively (Table 2). In the subgroup analysis stratified by characteristics of CVD mortality, only the difference in surgical status was not significant (*P* = 0.08), while other characteristics showed significant intergroup differences (*P* < 0.001). The CIF of death due to BC gradually slowed down with survival time, while the CIF of death due to CVD steadily increased. Among non-BC causes of death, cardiovascular deaths are almost comparable to those from all other causes (Figure 1). In a subsequent subgroup analysis stratified by patients’ characteristics, we observed that a high CIF of death due to CVD occurred predominantly in patients aged ≥65 years, who were white, unmarried, and had grade I-II of the tumor, *in situ* or localized summary stage, and who were not receiving radiotherapy or chemotherapy (Figure 2).

**Table 2:** Cumulative incidence of cause-specific death and Gray's test in the whole cohort.

Characteristics	Death from CVD (%)		*P*	Death from BC (%)		*P*	Death from non-CVD (%)		*P*
	5-years	10-years		5-years	10-years		5-years	10-years	
Total	7.8	13.5		21.3	24.8		8.3	15.1	
Age (years)			<0.001			<0.001			<0.001
<65	2.0	3.9		15.9	18.2		3.1	5.8	
65–74	5.6	11.0		18.5	22.2		7.0	14.1	
75–84	11.7	20.8		24.8	29.3		12.0	22.9	
≥85	20.4	29.4		35.4	38.7		17.5	25.4	
Sex			<0.001			<0.001			<0.001
Male	8.3	14.3		19.8	23.3		8.6	15.5	
Female	6.4	11.3		25.8	28.8		7.5	13.9	
Race			<0.001			<0.001			<0.001
White	8.0	13.8		20.7	24.1		8.4	15.3	
Black	6.9	11.0		33.3	37.1		7.7	12.7	
Others	5.6	10.1		20.7	23.9		7.2	13.0	
Marital status			<0.001			<0.001			<0.001
Married	7.0	12.4		17.9	21.3		7.2	13.7	
Single/other	9.4	15.3		27.1	30.6		10.3	17.4	
Primary site			<0.001			<0.001			<0.001
C67.0-Trigone of bladder	7.5	13.7		20.8	23.8		7.7	14.0	
C67.1-Dome of bladder	8.4	14.2		27.1	30.7		8.5	15.6	
C67.2-Lateral wall of bladder	7.4	13.7		14.9	17.8		8.3	15.0	
C67.3-Anterior wall of bladder	9.0	15.4		26.2	29.5		9.6	16.1	
C67.4-Posterior wall of bladder	8.6	15.1		16.4	19.6		8.7	16.4	
C67.5-Bladder neck	8.8	14.6		24.3	27.2		8.1	14.1	
C67.6-Ureteric orifice	6.9	13.0		10.3	13.1		7.3	14.4	
C67.7-Urachus	2.4	3.2		37.3	45.5		4.1	6.4	
C67.8-Overlapping lesion of bladder	8.2	12.9		32.5	36.3		8.7	14.8	
C67.9-Bladder, NOS	7.8	12.9		23.0	26.9		8.3	15.2	
Histologic type			<0.001			<0.001			<0.001
Transitional cell carcinoma	8.0	13.7		19.9	23.4		8.4	15.3	
Squamous cell carcinoma	5.6	8.7		51.6	53.5		8.0	12.2	
Adenocarcinoma	5.6	7.8		52.1	57.1		7.0	8.3	
Other and Unclassified carcinoma	5.1	7.2		60.1	63.4		6.6	9.8	
Tumor grade			<0.001			<0.001			<0.001
I	7.4	14.2		4.3	6.7		8.1	16.4	
II	7.8	14.6		6.8	9.9		8.1	16.1	
III	8.4	13.3		34.9	38.8		8.8	14.5	
IV	7.7	12.2		33.9	38.2		8.3	13.9	
Summary stage			<0.001			<0.001			<0.001
In situ	7.8	14.9		3.6	6.1		8.2	16.6	
Localized	8.9	14.2		26.8	31.6		9.4	15.7	
Regional	5.4	7.6		60.1	63.9		6.5	9.2	
Distant	3.1	3.4		87.4	88.5		4.3	4.9	
Surgery			0.08			<0.001			0.03
No	8.3	12.7		34.7	36.8		8.4	13.6	
Yes	7.8	13.5		20.8	24.3		8.3	15.2	
Radiotherapy			<0.001			<0.001			<0.001
No	7.8	13.6		18.7	22.1		8.3	15.3	
Yes	8.8	11.5		63.0	66.9		8.8	11.9	
Chemotherapy			<0.001			<0.001			<0.001
No	8.5	14.4		17.9	21.2		8.8	15.9	
Yes	5.4	9.8		34.2	38.6		6.4	11.7	

### 3.3. Independent risk factors of death from CVD

According to the results of univariate analysis based on the Fine-Gray hazard model in the training cohort, age, sex, race, marital status, primary site, histologic type, tumor grade, summary stage, radiotherapy, and chemotherapy were significantly associated with CVD-related death (*p* < 0.05). Then, variables with a *p*-value < 0.05 during univariate analysis were selected for multivariate competing risk analysis to minimize the risks of producing false positive results. The analysis revealed that the following eight factors were independently associated with an elevated risk of cardiovascular death: age, sex, race, marital status, histologic type, tumor grade, summary stage, and chemotherapy (Table 3).

**Table 3:** Univariate and multivariable competing risk analysis for CVD-related death among patients with BC in the training cohort.

Variables	Univariate analysis		Multivariate analysis	
	HR (95% CI)	*P* value	HR (95% CI)	*P* value
Age (years)				
<65	Reference		Reference	
65–74	2.82 (2.64–3.02)	<0.001	2.89 (2.70–3.10)	<0.001
75–84	5.40 (5.07–5.74)	<0.001	5.55 (5.22–5.91)	<0.001
≥85	7.73 (7.23–8.27)	<0.001	8.00 (7.45–8.55)	<0.001
Sex				
Male	Reference		Reference	
Female	0.79 (0.76–0.83)	<0.001	0.68 (0.65–0.71)	<0.001
Race				
White	Reference		Reference	
Black	0.79 (0.71–0.85)	<0.001	0.97 (0.88–1.06)	0.527
Others	0.76 (0.69–0.84)	<0.001	0.78 (0.71–0.86)	<0.001
Marital status				
Married	Reference		Reference	
Single/other	1.25 (1.20–1.29)	<0.001	1.22 (1.17–1.27)	<0.001
Primary site				
C67.0-Trigone of bladder	Reference		Reference	
C67.1-Dome of bladder	1.08 (0.96,1.22)	0.185	1.02 (0.90–1.14)	0.789
C67.2-Lateral wall of bladder	1.07 (0.98,1.15)	0.123	1.04 (0.96–1.12)	0.392
C67.3-Anterior wall of bladder	1.17 (1.02,1.34)	0.021	1.09 (0.95–1.25)	0.213
C67.4-Posterior wall of bladder	1.14 (1.04,1.25)	0.005	1.05 (0.96–1.15)	0.318
C67.5-Bladder neck	1.08 (0.95,1.23)	0.237	1.03 (0.90–1.17)	0.695
C67.6-Ureteric orifice	1.03 (0.92,1.15)	0.613	1.03 (0.92–1.15)	0.656
C67.7-Urachus	0.21 (0.10,0.56)	0.002	0.73 (0.27–1.94)	0.524
C67.8-Overlapping lesion of bladder	1.00 (0.91,1.09)	0.996	1.01 (0.92–1.10)	0.891
C67.9-Bladder, NOS	0.99 (0.91,1.07)	0.757	0.96 (0.89–1.04)	0.311
Histologic type				
Transitional cell carcinoma	Reference		Reference	
Squamous cell carcinoma	0.60 (0.51,0.71)	<0.001	0.73 (0.61–0.86)	<0.001
Adenocarcinoma	0.46 (0.34,0.62)	<0.001	0.71 (0.52–0.96)	0.028
Other and unclassified carcinoma	0.53 (0.43,0.65)	<0.001	0.82 (0.67–1.01)	0.064
Tumor grade				
I	Reference		Reference	
II	1.02 (0.97,1.08)	0.459	0.99 (0.94–1.04)	0.705
III	0.95 (0.89,1.00)	0.071	0.92 (0.86–0.98)	0.009
IV	0.79 (0.75,0.84)	<0.001	0.77 (0.72–0.82)	<0.001
Summary stage				
In situ	Reference		Reference	
Localized	0.95 (0.91,0.98)	0.004	0.90 (0.86–0.94)	<0.001
Regional	0.49 (0.45,0.53)	<0.001	0.60 (0.55–0.66)	<0.001
Distant	0.22 (0.19,0.27)	<0.001	0.29 (0.24–0.35)	<0.001
Surgery				
No	Reference			
Yes	1.07 (0.97,1.19)	0.183		
Radiotherapy				
No	Reference		Reference	
Yes	0.82 (0.76,0.90)	<0.001	0.96 (0.87–1.05)	0.344
Chemotherapy				
No	Reference		Reference	
Yes	0.57 (0.54,0.60)	<0.001	0.77 (0.73–0.82)	<0.001

### 3.4. Construction of the competing risk model

**Figure 3:**
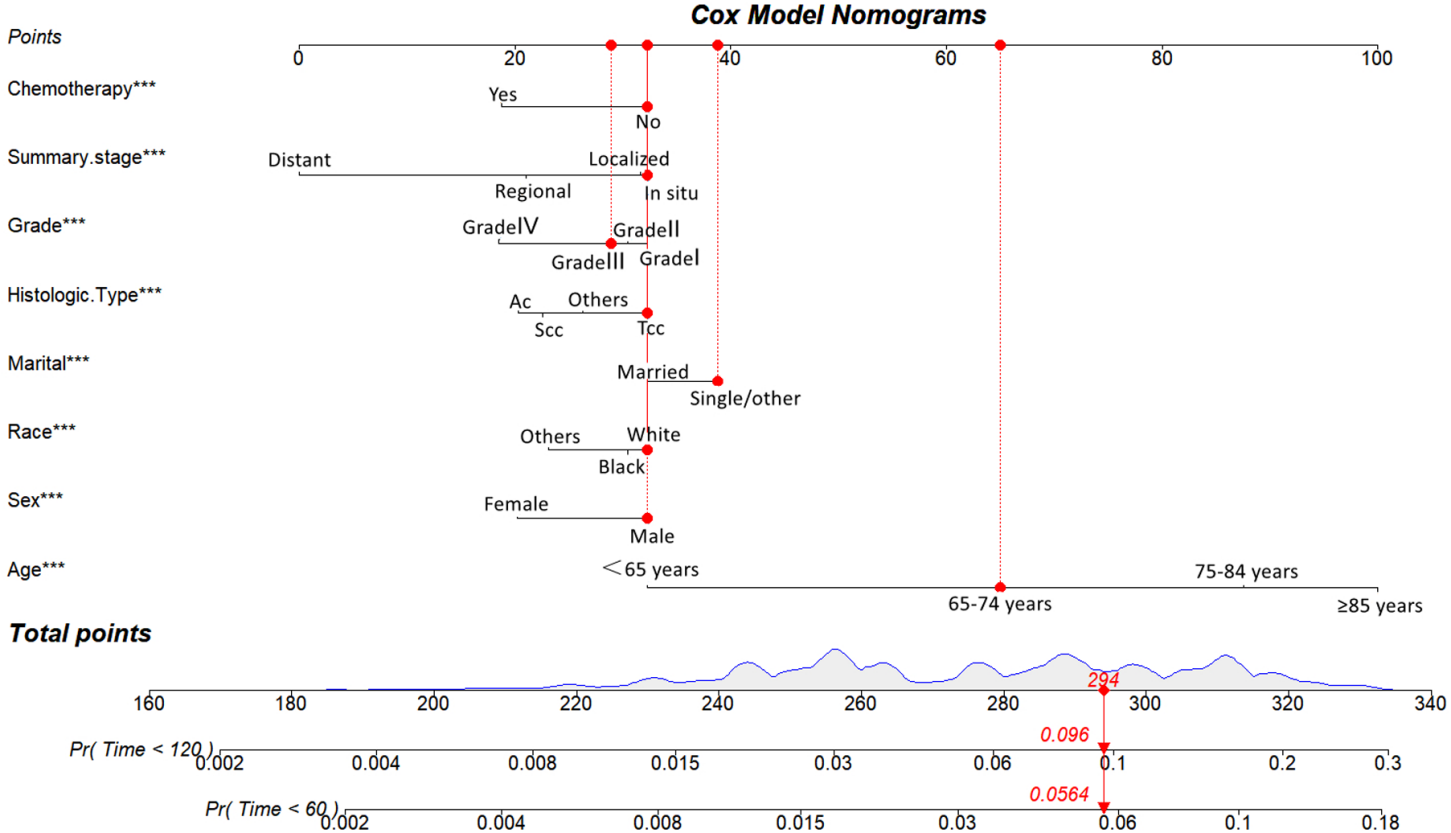
Competing risk model for predicting the 5-, and 10-year probabilities of CVD-related death among patients with BC. For example, for an 68-year-old married white race male patient with grade III, Tcc of histologic type, *in situ* of summary stage of BC that did not undergo chemotherapy, the total score is 65(68 years old) + 32(male) + 32 (white race) + 39 (single/other) + 32(Tcc) + 30(Grade III) + 32 (*in situ*) + 32(no chemothaoy)= 294, and the corresponding risk of CVD-related death at 5- and 10-year are 0.0564 and 0.096.

Utilizing the eight identified factors, a nomogram was established for predicting long-term CVD-related death in BC patients based on the Fine-Gray competing risk model. As illustrated in Figure 3, the corresponding point value of each independent risk factor in the nomogram was obtained by drawing a straight line to the top point row and then summed to obtain the total point. By drawing vertical lines from the total point row to the bottom timeline, it is possible to estimate the likelihood of cardiovascular mortality in patients with BC based on their individual characteristics.

### 3.5. Validation of the competing risk model

**Figure 4:**
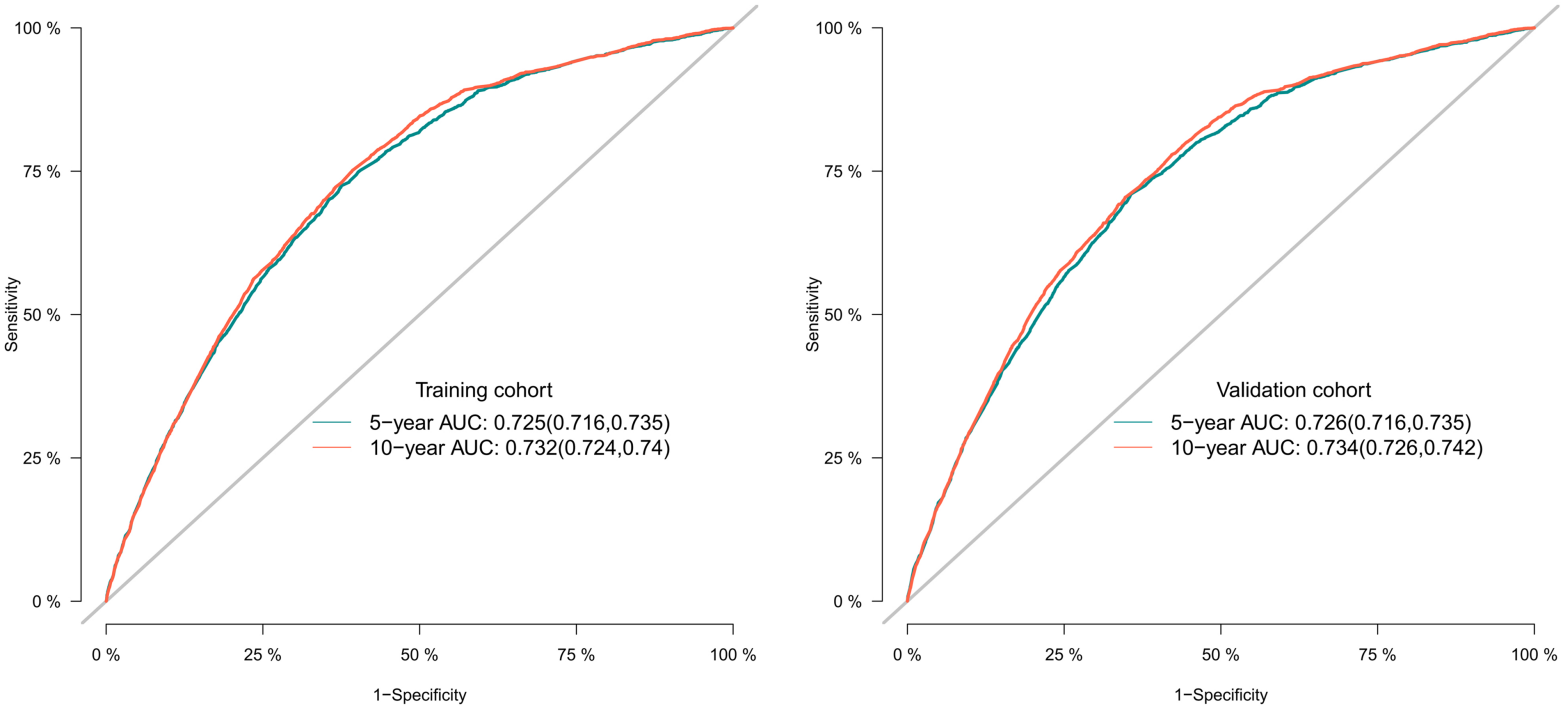
The receiver operating characteristic curves for predicting the 5-, and 10-year probabilities of CVD-related death in the training and the validation cohort, respectively.

**Figure 5:**
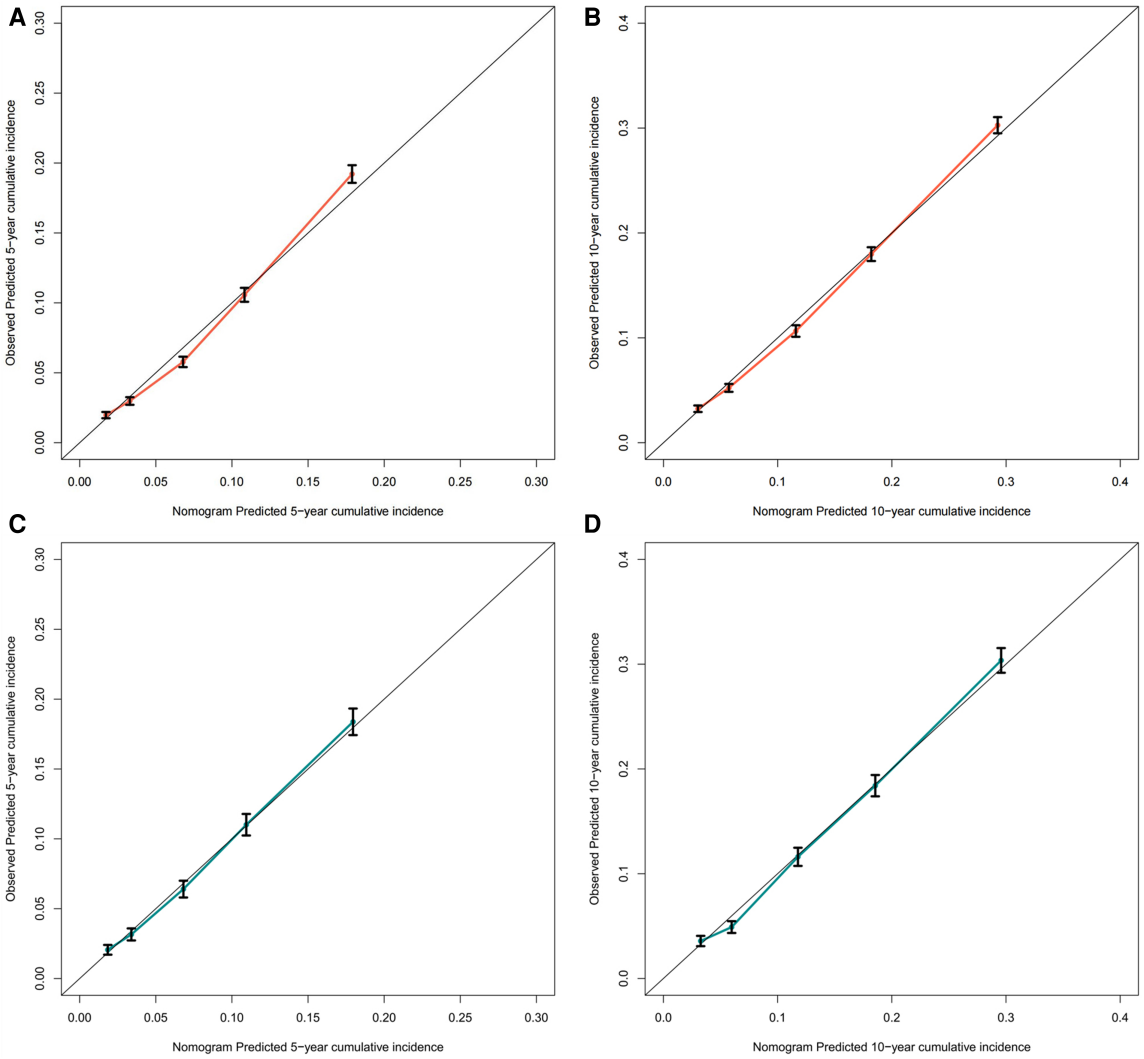
The calibration curve for predicting the 5-, and 10-year probabilities of CVD-related death in the training (**A,B**) and the validation cohort (**C,D**), respectively.

**Figure 6:**
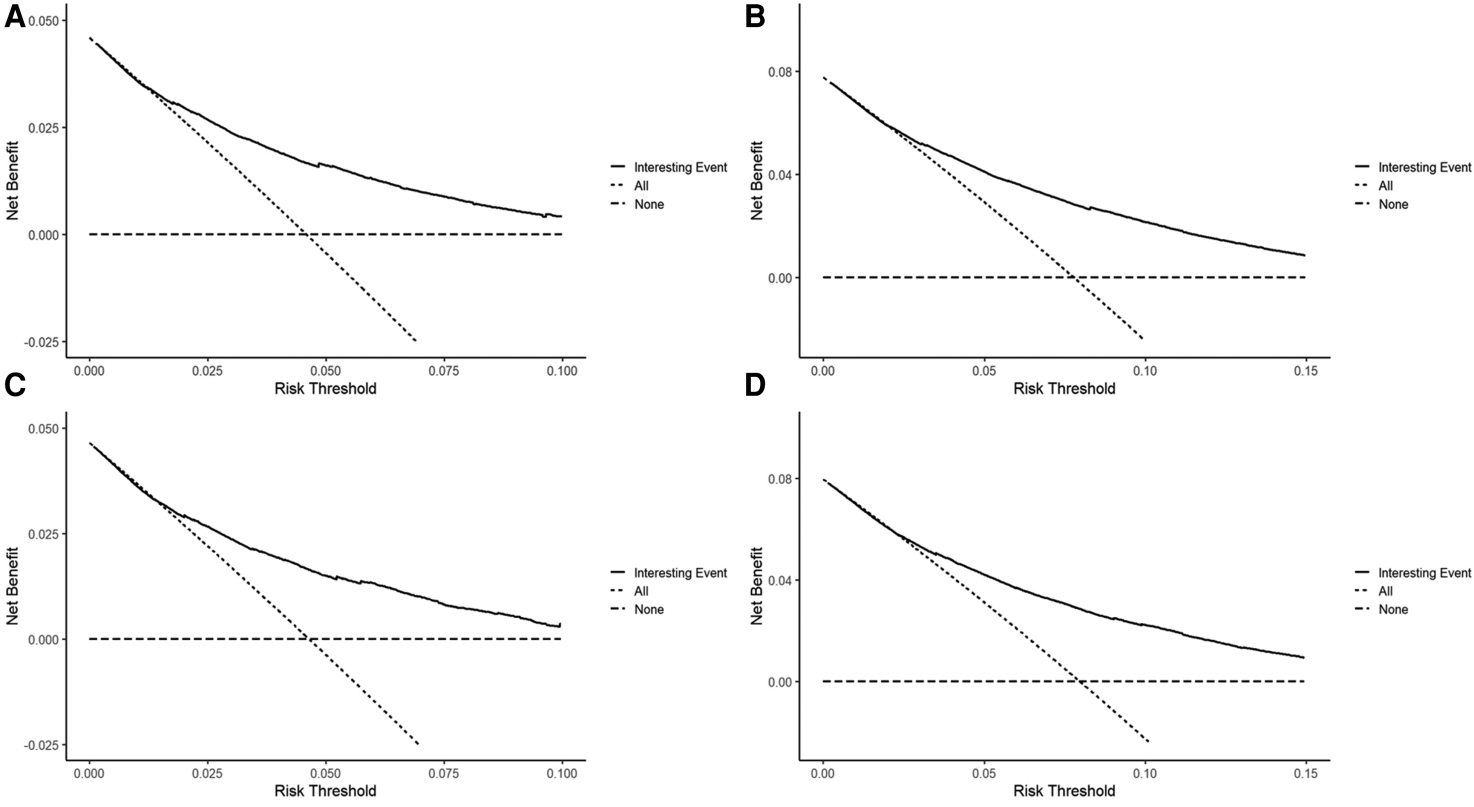
The decision curves for predicting the 5-, and 10-year probabilities of CVD-related death in the training (**A,B**) and the validation cohort (**C,D**), respectively.

To assess the discriminative performance of the model, we calculated the bootstrap-corrected C-index, which was 0.716 and 0.709 in the training cohort and validation cohort, respectively. While the AUCs for the 5-year probabilities of CVD in the training and validation cohorts were 0.725 and 0.732, respectively. Consistently, the AUCs for the 10-year probabilities of CVD in the training and validation cohorts were 0.726 and 0.734, respectively (Figure 4). These results showed that our model had a great discrimination ability in predicting CVD-related death in BC patients. Furthermore, we utilized the calibration curves to evaluate the accuracy of the nomogram and observed excellent consistency between the predicted probability of CVD-related death at 5- and 10-years and the actual observations in both the training and validation cohorts (Figure 5). Then, the DCA curves also revealed that the nomogram had high positive net benefits, indicating greater clinical application value and prospects in predicting CVD-related death in BC patients (Figure 6).

## 4. Discussion

In this large-scale, population-based study, we conducted a comprehensive assessment of cardiovascular mortality risk among 129765 patients with BC based on the SEER database. Overall 68,655 BC patients died during the follow-up period, and more than half of them (53.6%) died from non-tumor causes, with CVD-related death accounting for 46.7%. Furthermore, we identified age, sex, race, marital status, histologic type, tumor grade, summary stage, and chemotherapy as independent risk factors that were used to establish and validate a nomogram for predicting the 5-, and 10-year probabilities of CVD-related death in BC patients. To the best of our knowledge, this was the first study to develop a novel nomogram using the Fine-Gray competing risk model for predicting long-term cardiovascular mortality risk in BC patients.

The risk of cancer and CVD advance with age (21). Indeed, a prior investigation has demonstrated that BC patients ≥65 years may face a greater risk of CVD-related mortality compared to mortality attributed directly to BC (8). In addition, it is widely acknowledged that older patients generally have a worse prognosis, which is often attributed to a range of factors including reduced physical function, cognitive impairment, and comorbidities such as hypertension, hyperlipidemia, and diabetes. These factors can also contribute to an increased risk of CVD-related mortality in this patient population. Furthermore, we demonstrated that marital status can also potentially influence CVD-related death among BC survivors. Marriage offers a direct form of social support and greater financial resources, which can help to reduce the likelihood of engaging in unhealthy behaviors such as poor diet or alcohol use, and increase the probability of receiving timely screening facilities and medical care (22–24). Conversely, unmarried individuals may have less access to emotional, financial, and companionship support, and may even experience more sub-clinical symptoms of depression, anxiety, and major mental disorders, all of which can contribute to an increased risk of cardiovascular mortality when compared to their married counterparts (23, 25–27).

Recent efforts have identified sex and racial disparities in BC (28, 29). It is also worth mentioning that BC is one of the top nonreproductive cancers exhibiting stark male and female differences: men are at a 3–5 times greater risk of developing BC, while females are more likely to be diagnosed with advanced-stage disease (30). Our results also show that the 5-, and 10-year CIF of BC were 19.8 and 23.3% in males, 25.8 and 28.8% in females. While, the 5-, and 10-year CIF of CVD were 8.3 and 14.3% in males, and 6.4 and 11.3% in females, indicating that female patients may be more likely to die from BC and male patients may be more likely to die from CVD. The observed sex disparities in mortality rates may be partially explained by unequal smoking patterns between males and females (31–33). Interestingly, this difference is also observed across ethnic groups. Despite BC incidence rates being two times higher in White people than in Black people, the latter experience a higher tumor stage at presentation and worse cancer-specific survival (34, 35). Possible contributing factors may include delayed diagnosis, surgical treatment at low-volume hospitals, and poor adherence to evidence-based care among Black people (36, 37).

Another independent risk factor of CVD-related death in BC patients is transitional cell carcinoma (TCC) of histologic type (17). TCC is the most prevalent histological subtype of BC and approximately 80% are non-muscle invasive at the time of diagnosis (38). The other histological subtype of BC, such as squamous cell carcinoma (SCC) and adenocarcinoma (AC), are much less common, all of which often present at an advanced stage and have shown a poor prognosis (39). A previous study showed that risk factors for SCC include schistosomiasis, chronic inflammation, recurrent urinary tract infections, and prior exposure to cyclophosphamide chemotherapy (40). While environmental exposures such as cigarette smoke and occupational exposures are the most well-established risk factors for TCC. Thus smoking has been shown to further contribute to the development of CVD in patients with TCC, through increased heart rate and myocardial contraction, inflammation, endothelial damage, thrombosis, and decreased serum HDL cholesterol levels (41–44).

Moreover, we observed that the CVD-related death of BC patients with Grade I/II and *in situ* or localized stage was higher than those with Grade III/IV and regional or distant stage. On the one hand, low-grade tumors have a low progression rate and can be initially treated with endoscopic approaches and surveillance but are generally not life-threatening to the patient. On the other hand, high-grade tumors have a high malignant potential and are associated with significant rates of progression and cancer-related mortality (45). The 5-, and 10-year CIF of BC were 3.6 and 6.1% in the stage of *in situ*, 87.4 and 88.5% in the stage of distant, providing compelling evidence for the critical need to improve early diagnosis rates of BC.

In terms of treatment, chemotherapy is widely used for BC which included systemic chemotherapy, neoadjuvant chemotherapy, and local perfusion chemotherapy. The current first-line chemotherapy drugs recommended by multiple treatment guidelines, such as cisplatin, anthracycline, mitomycin, and epirubicin magnitude, all come with varying degrees of cardiovascular toxicity, which significantly increases the risk of cardiovascular mortality (46). However, our study yielded a seemingly contradictory finding that the BC patients with chemotherapy were at a lower risk of CVD compared with those without chemotherapy. Notably, this result is consistent with previous studies conducted on other tumor types, such as colorectal cancer (47) and primary central nervous system lymphoma (48). The reasons for this association could be related to the following factors. Firstly, there were several instances of missing records for treatment data in the SEER database, including the information on patient's chemotherapy regimen and duration, as well as the presence of co-existing CVD at the time of diagnosis. Individuals with baseline CVD have contraindications to chemotherapy, and subjects with better cardiovascular status are more likely to receive chemotherapy (49). Therefore, a higher risk of cardiovascular death was observed in those not receiving chemotherapy. Secondly, chemotherapy is generally used for muscle-invasive BC with a relatively high stage, so limited life expectancy and a greater likelihood of death from BC. The underlying mechanism remains unclear and further investigation is needed. Therefore, these results should be interpreted with caution. However, it is noteworthy that some potential new strategies, such as gliflozins, have been shown to possess anti-inflammatory and cardioprotective effects against anthracycline-induced cardiotoxicity, thus may offering a new treatment option for chemotherapy patients (50).

The strengths of our study were that this was the first study to develop a novel nomogram for predicting long-term cardiovascular mortality risk in patients with BC based on the competing risk model. Furthermore, our study benefited from a large enough sample size and long-term follow-up based on the SEER database, which covers a significant proportion of the US population (approximately one-third). Then, given the potential presence of competing events in the analysis of CVD through the Cox regression model, we utilized a competing risk model to investigate risk factors and develop a novel nomogram. Moreover, our nomogram showed good discrimination power, excellent consistency, and clinical practicability.

There are still some limitations in our study. First of all, even though our analysis is computer-based, the underlying data is sourced from the SEER database which records real-world patients from high-quality cancer registries and relies on systematic, standardized, and regular data collection procedures for quality assurance and the avoidance of surveillance bias (51). Second, given that our study is a retrospective cohort study based on the SEER database, selection bias is inevitable. Third, the SEER database did not provide detailed information on cardiovascular comorbidities or other relevant cardiovascular-related factors, such as blood pressure, blood glucose, smoking, alcohol consumption, and so on. This limitation precluded a more comprehensive investigation into their potential impact on the risk of CVD mortality. Fourth, data on specific regimens and duration of chemotherapy or immunotherapy were not recorded in the SEER database, despite these factors being closely linked to the development of cardiotoxicity. Lastly, it is important to acknowledge that our competing risk model was developed using risk factors that were solely recorded in the SEER database and did not account for other potentially relevant risk factors that were not recorded. Therefore, while the model demonstrated promising results, external validation is still necessary to further confirm its utility and reliability in clinical practice.

## 5. Conclusion

Given that BC patients have the highest risk of dying from CVD among 28 cancer types, it is imperative to enhance the management of CVD in this patient population. Our study has identified several independent risk factors and built a novel nomogram using a competing risk model to accurately predict the probabilities of long-term cardiovascular mortality in BC patients. With the help of this well-established nomogram, urologists and cardiologists could work together to comprehensively assess the potential risks of treatment, providing early monitoring and prevention of heart damage, thereby minimizing factors that are harmful to a patient's cardiovascular prognosis.

## Data Availability

The original contributions presented in the study are included in the article/Supplementary Material, further inquiries can be directed to the corresponding author.
